# Integration of smart sensors and phytoremediation for real-time pollution monitoring and ecological restoration in agricultural waste management

**DOI:** 10.3389/fpls.2025.1550302

**Published:** 2025-05-13

**Authors:** Jinsong Guo, Xiaoxin Lin, Yingjun Xiao

**Affiliations:** ^1^ School of Economics, Guangdong Ocean University, Zhanjiang, China; ^2^ School of Mathematics and Computer Science, Guangdong Ocean University, Zhanjiang, China

**Keywords:** 3D reconstruction, landscape restoration, hybrid method, point cloud, ecological integrity, attention mechanism, graph networks

## Abstract

Global climate change and ecological degradation highlight the urgency of dealing with agricultural waste and ecological restoration. Traditional pollutant monitoring and ecological restoration methods face challenges in accuracy and adaptability, especially when dealing with complex environmental data. This paper proposes the Bio-DANN model, which combines biogeochemical models and deep learning techniques to improve the accuracy of pollutant monitoring and ecological restoration prediction. The model uses deep neural networks (DNNs) and attention mechanisms to process multidimensional environmental data in various agricultural and ecological scenarios in real time. Experimental results based on Open Soil Data and NEON datasets show that Bio-DANN performs well in pollutant prediction, with mean square errors (MSE) of 0.012 and 0.018, root mean square errors (RMSE) of 0.109 and 0.134, and accuracy of 0.92 and 0.90, respectively. In terms of ecological restoration assessment, Bio-DANN achieved Δ*F* and PIPGR of 0.15 and 18%, and 0.20 and 22%, respectively, and H’ values of 1.5 and 1.7, which are better than other models. Bio-DANN provides a promising technical solution for environmental protection, resource recovery and sustainable agriculture, especially showing significant potential in pollutant monitoring, soil health assessment and ecological restoration evaluation.

## 1 Introduction

With the intensification of global climate change, population growth, and increasing environmental degradation, the management of agricultural waste and ecological restoration has become an urgent challenge (Shi et al., 2023). For instance, large-scale agricultural activities generate significant waste, contributing to soil pollution and ecosystem degradation. This is particularly evident in regions such as Southeast Asia and Sub-Saharan Africa, where agricultural runoff contaminates water sources and reduces soil fertility (Singh et al., 2024). In this process, pollutant monitoring and the evaluation of ecological restoration effectiveness remain key issues. As environmental problems become more severe, traditional pollutant treatment methods often suffer from poor real-time performance, insufficient accuracy, and weak adaptability to complex ecosystems (Singh et al., 2023). Phytoremediation, as an eco-friendly remediation approach, can effectively improve soil health and ecological environments by absorbing and transforming pollutants through plants (Singh et al., 2024). For example, the use of plants like poplar and willows in contaminated sites has shown promising results in removing heavy metals from soils (Gao et al., 2021). However, the dynamic changes in pollutants during the phytoremediation process are highly complex, and evaluating remediation effectiveness involves multiple dimensions (Naizabayeva et al., 2024; Wang and Delavar, 2024). Accurately and in real-time monitoring these changes remains a significant challenge in current research.

In recent years, AI-based pollutant monitoring and ecological evaluation methods have made some progress, particularly in integrating deep learning with sensor technologies. For example, the combination of deep learning models and IoT sensors has been used in some smart agriculture projects to monitor soil moisture and pollutant levels in real-time (Wu and Zhao, 2023). However, most existing approaches still struggle with accuracy limitations, poor real-time performance, and constrained data processing capabilities when dealing with complex biogeochemical processes. For example, many studies employ traditional CNNs to predict soil pollutant concentrations. While CNNs can achieve a certain level of predictive accuracy, they often fail to fully utilize temporal sequence data, resulting in lower accuracy for long-term predictions (Wu and Zhao, 2023). Additionally, studies leveraging Recurrent Neural Networks (RNNs) or Long Short-Term Memory (LSTM) models have made breakthroughs in handling time-series data, yet they still face limitations in modeling complex ecological processes, particularly in capturing multivariate relationships among pollutants, soil properties, and plant growth (Gao et al., 2021). Similarly, research on ecological restoration evaluation faces comparable challenges. Many existing studies use simple regression analysis or remote sensing-based statistical models to assess changes in soil fertility or biodiversity during phytoremediation (Gao et al., 2021). However, these methods often rely excessively on static data and lack the ability to track the dynamic changes of ecological restoration in real-time. For instance, some remote sensing-based soil fertility assessment models can periodically evaluate the spatial distribution of soil fertility but lack the capability to track the temporal dynamics of plant growth and soil changes, making them less effective in adapting to complex ecological variations.

To address these challenges, this study proposes the Bio-DANN model (Biogeochemical-Deep Attention Neural Network Model), which integrates biogeochemical models, deep neural networks (DNNs), and multi-head attention mechanisms to enable dynamic pollutant monitoring and ecological restoration evaluation in agricultural waste management. The Bio-DANN model builds on the foundational principles of biogeochemical models, which simulate the processes of pollutant absorption and transformation by plants in the soil (Singh et al., 2024). Unlike existing methods, Bio-DANN leverages deep attention mechanisms to effectively capture the impact of various ecological factors, such as temperature, rainfall, and soil composition, when processing high-dimensional, complex time-series data. This approach overcomes the limitations of traditional methods in handling dynamic changes in complex ecosystems. In particular, BioDANN demonstrates superior performance in predicting pollutant concentrations over multi-dimensional and long-term time scales, as well as in evaluating the effectiveness of ecological restoration. Through a combination of deep learning and biogeochemical modeling, Bio-DANN provides a more robust framework for predicting pollutant dynamics and assessing ecological restoration in real-time, offering promising potential for applications in smart agriculture and environmental management.

The main contributions of this paper are as follows:

- The introduction of the Bio-DANN model, which effectively combines traditional phytoremediation methods with modern deep learning techniques, filling a gap in existing research on the integration of phytoremediation and deep learning.
- The design of a deep learning framework based on biogeochemical processes, enabling the model to better simulate and predict the dynamic changes of pollutants during the phytoremediation process.
- Experimental verification demonstrating the high efficiency and accuracy of the Bio-DANN model in pollutant concentration prediction and ecological restoration evaluation, with particular potential for application in agricultural waste management.

The structure of this paper is as follows: Section 2 reviews the research progress on phytoremediation technology and pollutant management, as well as the application of deep learning in environmental monitoring. Section 3 provides a detailed introduction to the design and implementation of the Bio-DANN model, including the biogeochemical model, deep neural network module, multi-head attention mechanism, and the integrated output and evaluation module. Section 4 presents the selection of experimental datasets, experimental setup, and results analysis. Finally, Section 5 summarizes the research findings and discusses the future prospects of the Bio-DANN model in broader application areas.

## 2 Related work

### 2.1 Phytoremediation technology and pollutant management

Phytoremediation, as a natural ecological restoration method, has become a crucial approach for addressing soil and water pollution and restoring ecological environments (Wijekoon et al., 2021). By leveraging the absorption, transformation, and degradation capabilities of specific plants, this technique effectively reduces environmental pollution, improves soil quality, and restores the functionality of ecosystems. Compared with traditional physical and chemical remediation methods, phytoremediation offers distinct advantages such as environmental friendliness, cost-effectiveness, and strong sustainability (Agarwal and Rani, 2022). It has been extensively studied and applied in areas such as agricultural waste management, industrial wastewater treatment, and heavy metal contamination.

The fundamental mechanisms of phytoremediation can be broadly categorized into three main types: phytoextraction, phytodegradation, and phytostabilization. Phytoextraction involves the uptake of pollutants such as heavy metals, pesticides, or organic contaminants from soil or water into plant tissues through the root system (Almayyan and AlGhannam, 2024; DeMatteo et al., 2024; Zhang et al., 2025). Phytodegradation refers to the metabolic processes within plants that transform pollutants into harmless substances. Phytostabilization is the process by which plants secrete substances through their root systems to immobilize or precipitate pollutants, thereby reducing their bioavailability (Takkar et al., 2022). In recent years, as phytoremediation technology has evolved, researchers have increasingly recognized the limitations of relying on single plant species to address the complexity of various pollutants and environmental conditions. This has led to new research focuses, including the development of composite phytoremediation systems, plant-microbe symbiosis for enhanced remediation, and the application of genetically engineered plants (Phang et al., 2024).

Despite its promising potential, phytoremediation faces several challenges in practical applications. First, different plant species exhibit significant variability in their ability to absorb and degrade pollutants, making the selection of appropriate plant species a critical issue (Nedjimi, 2021). Second, the process of phytoremediation is time-consuming and often has low efficiency, particularly in environments with high pollution levels where the remediation capabilities of plants may not meet the requirements for rapid recovery (Park and Oh, 2023). Furthermore, environmental variables such as climate change, soil pH fluctuations, and water availability can significantly impact the effectiveness of phytoremediation. Therefore, while phytoremediation holds great promise, improving its efficiency and adaptability remains a pressing challenge.

With the rise of big data, artificial intelligence, and deep learning technologies, the research and application of phytoremediation are moving toward increased intelligence and efficiency. By utilizing environmental sensors to monitor dynamic pollutant changes and integrating these data with deep learning models to predict pollutant concentrations and remediation outcomes, the precision and timeliness of phytoremediation can be greatly enhanced (Tripathi et al., 2020). In the context of agricultural waste management, intelligent monitoring and remediation systems can effectively combine phytoremediation with dynamic pollutant management to improve remediation efficiency and reduce environmental risks. This paper innovatively integrates phytoremediation techniques with deep learning models to address issues such as low efficiency and slow response in traditional phytoremediation processes, thereby enhancing pollutant monitoring and ecological restoration in agricultural waste management.

### 2.2 Application of deep learning in environmental monitoring

Deep learning, with its powerful data processing capabilities and automated feature extraction abilities, has become an essential tool in the field of environmental monitoring. By training deep neural networks, deep learning can uncover hidden patterns and relationships from large-scale, complex environmental data, providing accurate decision support for pollutant monitoring, ecological restoration evaluation, and environmental quality prediction (Yuan et al., 2020; Wang et al., 2025b). In particular, deep learning excels in processing various types of time-series data, image data, and sensor data, significantly enhancing the real-time response to pollutant monitoring and environmental changes. In the area of pollutant monitoring, deep learning has been widely applied in the detection of pollutants in environmental media such as air, water, and soil. Traditional pollutant monitoring methods mainly rely on chemical analysis techniques (Ullo and Sinha, 2020; Wang et al., 2025a). While these methods can provide precise measurements, they are complex, costly, and unable to perform real-time monitoring. In contrast, deep learning models based on sensor data can predict pollutant concentrations with high frequency and precision and enable dynamic monitoring of environmental pollution through real-time data (Zhang et al., 2025a; Zhang et al., 2025b). For example, by analyzing data from air quality monitoring sensors using deep learning, it is possible to predict the trends of various pollutants (such as PM2.5, NOx, CO2, etc.) under different environmental conditions, providing valuable information for environmental management and policy-making (Ghannam and Techtmann, 2021).

Beyond pollutant monitoring, deep learning’s application in ecological restoration evaluation is also gaining increasing attention. Ecological restoration typically involves the interaction of multiple variables, such as soil health, plant growth, and microbial communities (Reddy et al., 2020; Tlijani et al., 2023; Ping and Yue, 2024). Traditional ecological models often struggle to efficiently process these complex, multidimensional data. Deep learning, with its powerful nonlinear mapping capabilities, can extract meaningful features from large amounts of environmental data and predict the outcomes of ecosystem restoration (Wu et al., 2021; Subramanian et al., 2023). For instance, by analyzing multidimensional data on soil quality, plant growth, and pollutant concentrations with deep neural networks, it is possible to more accurately assess the ecological changes occurring during the phytoremediation process, thus providing a scientific basis for optimizing restoration strategies (Dang et al., 2021). Currently, the application of deep learning in environmental monitoring is primarily focused on data analysis and predictive model development. However, integrating deep learning with biogeochemical models to simulate the transformation and degradation processes of pollutants, while conducting systematic evaluations of ecological restoration outcomes, remains a challenge that needs to be addressed (Kahl et al., 2021; Chamara et al., 2022). Although deep learning has shown strong predictive capabilities in environmental monitoring, existing research has largely concentrated on single-level pollutant detection and lacks comprehensive modeling of complex ecological processes. This limitation restricts the full application of deep learning in ecological environmental management.

Therefore, this paper combines biogeochemical models with deep learning technology, utilizing a multi-head attention mechanism to model dynamic pollutant changes. This not only improves the accuracy and timeliness of pollutant monitoring but also enhances the precision and comprehensiveness of ecological restoration evaluations. Through the development of this model, the gaps in current research are addressed, enabling the intelligent and efficient monitoring of pollutants and ecological restoration evaluation in agricultural waste management.

## 3 Methods

### 3.1 Overview of the Bio-DANN model architecture

**Figure 1:**
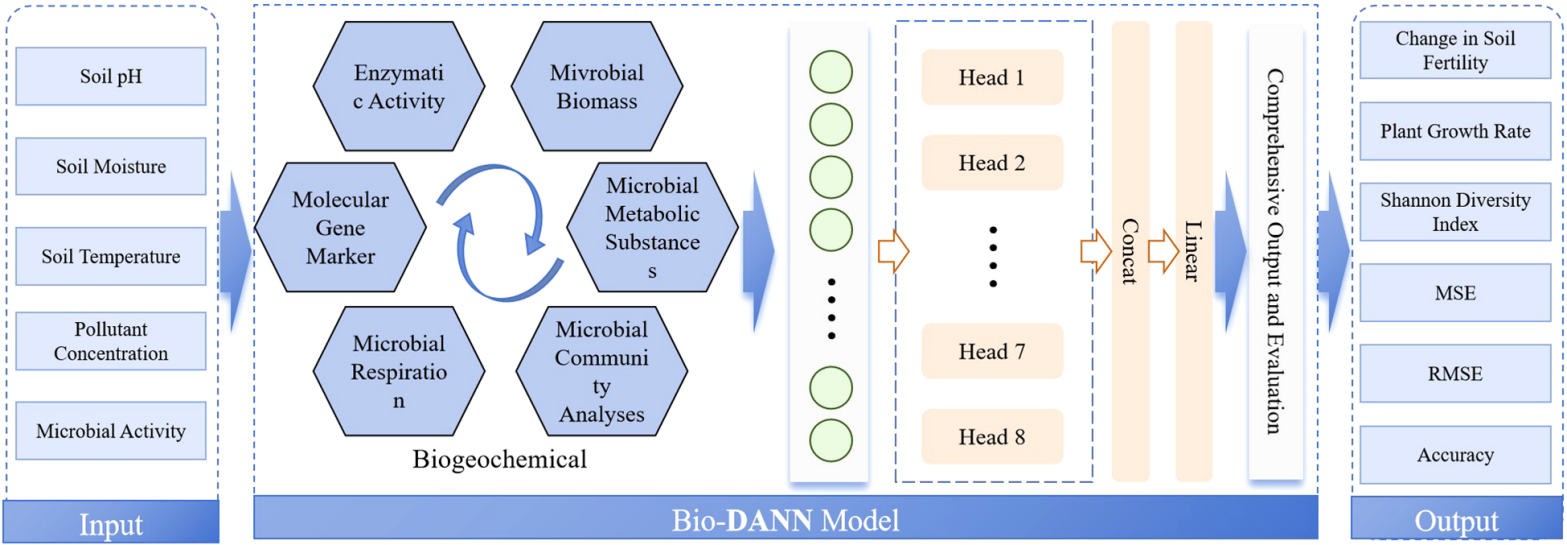
Schematic diagram of the Bio-DANN model architecture. The model centers around the biogeochemical model module, deep neural network module, and multi-head attention mechanism module, working in synergy to achieve dynamic pollutant monitoring and ecological restoration assessment.

The Bio-DANN model (Biogeochemical-Deep Attention Neural Network Model) is an integrated intelligent system composed of multiple modules designed to combine biogeochemical models, deep neural networks (DNNs), and multi-head attention mechanisms for pollutant monitoring and ecological restoration assessment in agricultural waste management. The overall structure of the model is illustrated in **Figure 1**. Through the collaborative operation of its various modules, Bio-DANN efficiently handles the complex data involved in agricultural waste management, accurately simulates the dynamic changes of pollutants, and provides real-time assessments of ecological restoration.

As depicted in **Figure 1**, the Bio-DANN model begins with the biogeochemical model module, which simulates the absorption, transformation, and degradation of pollutants during the phytoremediation process. Phytoremediation, a natural environmental management method, gradually removes harmful substances through the interactions between plants and pollutants in the soil and air. The biogeochemical model calculates the pollutant removal efficiency based on information such as the types and concentrations of soil pollutants and soil properties. This module provides foundational data support for the subsequent deep neural network and attention mechanism modules, ensuring that the simulation of the remediation process aligns closely with actual ecological conditions.

Building upon the biogeochemical model, Bio-DANN incorporates a deep neural network module to further enhance the prediction capabilities for dynamic pollutant changes. Deep neural networks are adept at handling complex nonlinear relationships and, by learning from sensor data (e.g., soil moisture, temperature, gas composition), they can predict trends in pollutant concentrations and assess the impact of phytoremediation on ecological restoration. The key advantage of this module lies in its robust learning ability, which extracts implicit patterns from multidimensional data, thereby not only improving the precision of pollutant monitoring but also supporting the evaluation of ecological restoration outcomes.

Enhancing the model’s performance is the multi-head attention mechanism module, which processes different data features through multiple attention heads. This is particularly effective in analyzing time-series data, significantly boosting the model’s responsiveness to dynamic changes. In agricultural waste management, pollutant concentration variations are typically influenced by multiple factors such as seasonal changes, climate fluctuations, and soil moisture levels. The interactions among these factors result in highly sequential and complex data. The multi-head attention mechanism improves accuracy by identifying key features in time-series data. Through this mechanism, Bio-DANN can accurately capture the dynamic changes in pollutant concentrations and key alterations during the phytoremediation process, providing real-time feedback for environmental management.

Finally, the comprehensive output and evaluation module of the Bio-DANN model integrates the results from all modules to deliver final predictions of pollutant concentrations and assessments of ecological restoration. This module not only aggregates outputs from the biogeochemical model and deep neural network but also incorporates the learning outcomes from the multi-head attention mechanism applied to time-series data, thereby offering a holistic evaluation of phytoremediation effectiveness. The assessment encompasses various dimensions of ecological restoration, including soil health, plant growth status, and microbial community activity. The core function of this module is to provide a comprehensive and accurate evaluation of ecological restoration in agricultural waste management, thereby offering a scientific basis for future decision-making.

Through such multi-module collaboration, the Bio-DANN model effectively processes complex data in the agricultural waste management process, accurately predicts changes in pollutant concentrations, and evaluates the outcomes of ecological restoration. The highly integrated structure of this model not only enables real-time monitoring of pollutant variations but also dynamically assesses the impact of phytoremediation on environmental recovery, providing intelligent and precise solutions for agricultural waste treatment.

### 3.2 Biogeochemical model module

**Figure 2:**
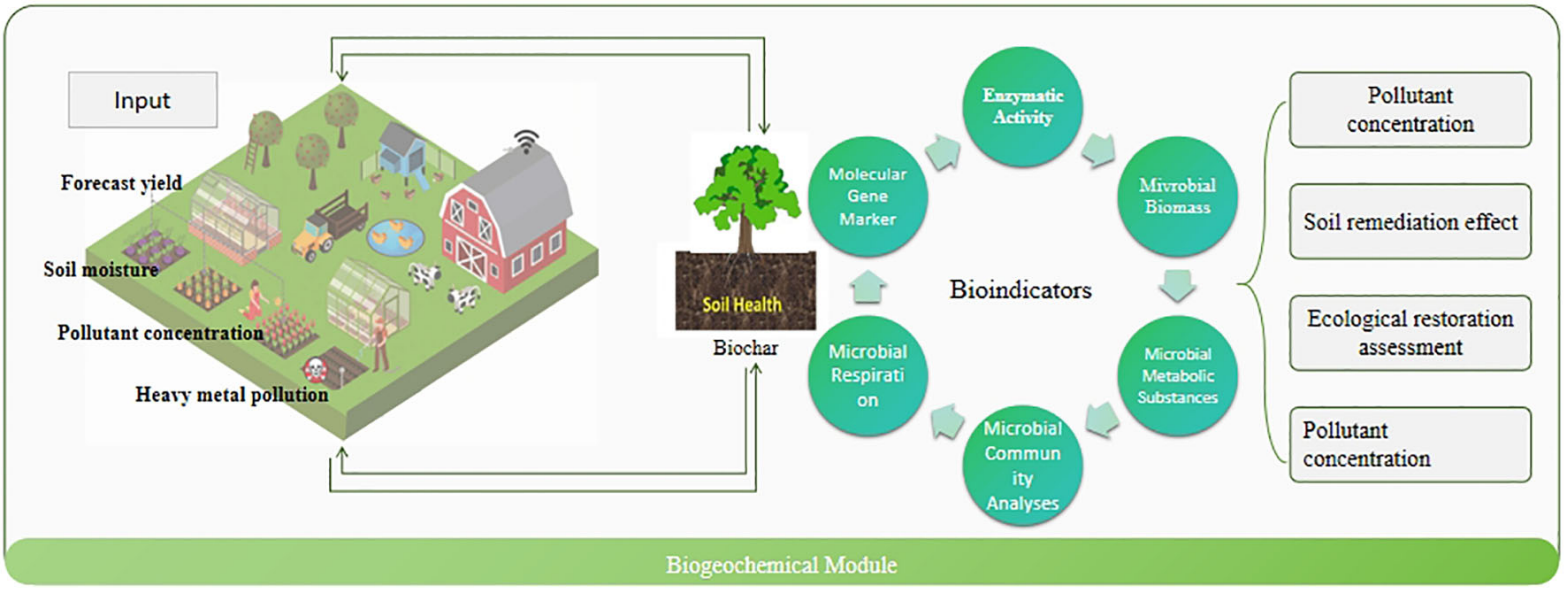
Architecture of the biogeochemical model module.

The biogeochemical model module is the core component of the Bio-DANN model, responsible for simulating the absorption, transformation, and degradation of pollutants during the phytoremediation process. This module integrates biogeochemical reaction mechanisms, utilizing mathematical models and reaction kinetics equations to predict pollutant removal efficiency and ecological restoration outcomes. It provides precise input data to the deep neural network module, facilitating subsequent pollutant dynamic monitoring and ecological restoration assessment. **Figure 2** illustrates the structure of the biogeochemical model module. This module simulates the transformation of pollutants in the soil and the absorption process by plants, ultimately evaluating the contribution of phytoremediation to ecological restoration. The overall design of the module consists of four main parts: pollutant input, pollutant transformation, plant absorption, and soil remediation effects.

At the initial stage of the model, the types and concentrations of pollutants are input into the system. Assuming the initial concentration of pollutants is *C*_0_, this value represents the initial concentration of pollutants in the soil (units: mg/kg). Pollutant input not only includes the type and concentration of pollutants but also considers environmental factors such as soil properties and climatic conditions, which influence the dynamic changes of pollutants.

The transformation process of pollutants in the soil is the core aspect of the model. Pollutants undergo a series of biogeochemical reactions, including redox reactions, hydrolysis reactions, and precipitation reactions, gradually transforming into harmless substances (Yang et al., 2022; Zhang et al., 2024). To simulate the degradation process of pollutants, the model employs reaction kinetics equations, assuming a reaction rate constant *k* that depends on soil type, temperature, humidity, and other environmental conditions (Kotiyal and Gupta, 2024). The change in pollutant concentration can be described by the following kinetic equation:


$$\frac{dC}{dt}=-k\cdot C$$

where *C*(*t*) represents the concentration of pollutants at time *t*, $\frac{dC}{dt}$ denotes the rate of change of pollutant concentration over time, and *k* is the reaction rate constant. This equation describes the transformation and disappearance of pollutants in the soil.

Phytoremediation is one of the primary pathways for pollutant removal. In the model, plants absorb pollutants through their root systems, incorporating them into the plant tissues. The amount of pollutants absorbed by plants, *M_p_*, is influenced by multiple factors, including plant growth status, soil pollutant concentration, and root characteristics. Assuming the plant absorption rate is *r_p_*, the amount of pollutants absorbed by plants can be expressed as:


$$M_{p}=r_{p}\cdot C\left(t\right)$$

where *M_p_* is the amount of pollutants absorbed by plants, *r_p_* is the plant absorption rate, and *C*(*t*) is the concentration of pollutants. The absorption rate *r_p_* is closely related to the plant’s growth status; therefore, the model incorporates plant growth indices (such as leaf area index or plant biomass) to dynamically adjust the absorption rate.

In addition to direct absorption, plants may also transform pollutants through metabolic processes. For example, some pollutants are metabolized into harmless substances within the plant or released into the atmosphere through transpiration. This transformation process is also accounted for in the model. Assuming the transformation rate of pollutants is *k_t_*, the kinetic equation for pollutant transformation within plants can be represented as:


$$\frac{dM_{p}}{dt}=k_{t}\cdot M_{p}$$

where *M_p_* is the amount of pollutants within the plant, and *k_t_* is the transformation rate constant. This equation allows the model to simulate the metabolic transformation of pollutants within the plant tissues.

Finally, the biogeochemical model module assesses the overall impact of the phytoremediation process on soil health. Soil remediation effects are primarily reflected in the reduction of pollutant concentrations, improvement of soil health, and restoration of soil microbial communities. As phytoremediation progresses, pollutant concentrations in the soil gradually decrease, and soil health improves. This process is demonstrated through changes in pollutant concentrations and the recovery of microbial activity in the soil.

By simulating the transformation processes of pollutants in the soil and the dynamic characteristics of phytoremediation, the biogeochemical model module can accurately evaluate the pollutant removal effectiveness of phytoremediation in agricultural waste management. This module not only considers the changes of individual pollutants but also integrates various environmental factors influencing remediation outcomes, providing reliable data support for the subsequent deep neural network module and multi-head attention mechanism module.

### 3.3 Deep Neural Network module (DNN)

**Figure 3:**
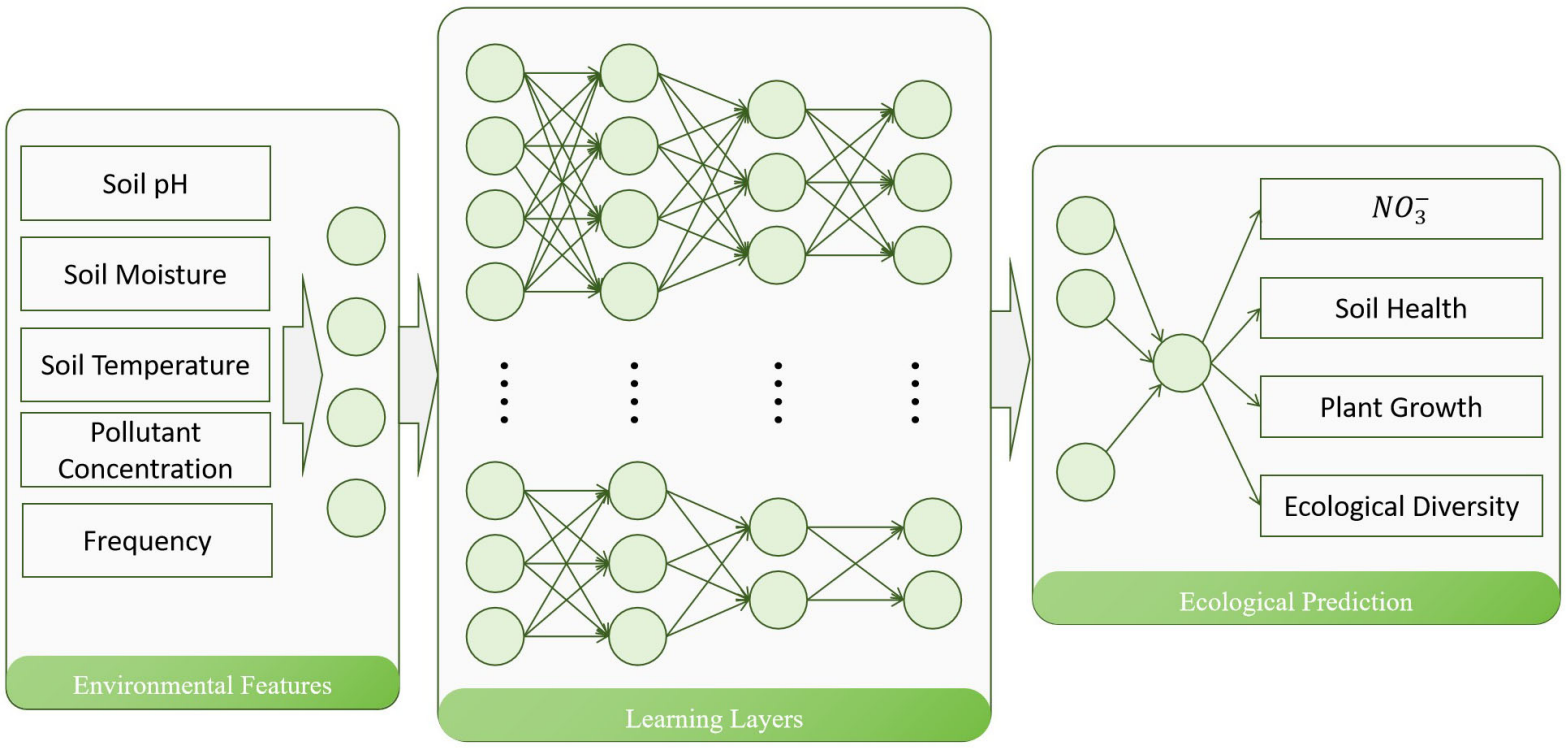
Architecture of the Deep Neural Network module. This diagram displays the multilayer structure of the neural network and the information transmission process.

In the Bio-DANN model, the Deep Neural Network (DNN) module plays a crucial role by extracting complex nonlinear patterns from environmental sensor data and predicting the dynamic changes in pollutant concentrations and ecological restoration outcomes based on these patterns. Through this module, the model can deeply explore the intricate relationships between pollutant concentrations and environmental variables, adjusting pollutant predictions in real-time to provide precise support for ecological restoration assessments. **Figure 3** illustrates the architecture of the Deep Neural Network module. This module consists of multiple fully connected layers, each of which transmits information to the next layer through weighted sums and bias operations. After processing with nonlinear activation functions, high-dimensional features of the environmental data are extracted layer by layer, ultimately outputting predictions for pollutant concentrations and restoration effects.

The core of the DNN module is a multilayer neural network, where each layer exchanges information with the preceding layer. Through successive nonlinear transformations, the network progressively extracts features from the input data (Dai et al., 2022; Radočaj et al., 2023). Let the input to the *l*-th layer be **x** *^l^*, and the output of that layer be **y** *^l^*. The computation for each layer can be represented as:


$$y^{l}=f\left(W^{l}x^{l-1}+b^{l}\right)$$

where **W** *^l^* is the weight matrix of the *l*-th layer, **b** *^l^* is the bias term, and *f*(·) is the activation function, typically ReLU (Rectified Linear Unit) or Sigmoid. **x** *^l^* ^−1^ is the output from the previous layer. Through this layer-by-layer information transmission and feature extraction, the network learns the complex relationships hidden within the input data, effectively capturing the dynamic changes in pollutant concentrations and ecological restoration.

In the Bio-DANN model, the DNN module receives input data from both the biogeochemical model and environmental sensor modules. These inputs include pollutant concentrations, soil characteristics, climatic conditions, and plant growth status. Leveraging the multilayer structure of the neural network, the model can learn and integrate this data, ultimately predicting trends in pollutant concentrations and the progress of ecological restoration.

During the training of the DNN module, soil pollutant concentrations, climatic conditions, and plant growth data collected by environmental sensors are used as training data. After preprocessing and normalization, these data are fed into the neural network. The model parameters are optimized by minimizing the difference between the predicted values and the actual observed values. To enhance the model’s generalization ability, random noise is added to further enrich the training data.

The training process utilizes Gradient Descent to optimize the model’s parameters. Assuming the model’s loss function is *L*(*θ*), where *θ* represents all parameters within the network, the update rule is:


$$\theta^{t+1}=\theta^{t}-\eta \nabla_{\theta }L\left(\theta \right)$$

where *η* is the learning rate, controlling the step size of each update, and ∇*_θ_L*(*θ*) is the gradient of the loss function with respect to the parameters. Through the backpropagation algorithm, the weights and biases of the neural network are continuously adjusted to reduce the loss function, thereby improving the model’s prediction accuracy.

Once the network is trained, the DNN module can predict pollutant concentrations and ecological restoration outcomes based on real-time sensor data. Input data (such as pollutant concentrations, soil conditions, and plant growth status) are processed through the neural network, and the output consists of predicted values for pollutant concentrations and restoration effects. Through the DNN module, the Bio-DANN model can dynamically adjust pollutant management strategies, providing precise and real-time decision support for agricultural waste treatment.

### 3.4 Multi-head attention module

**Figure 4:**
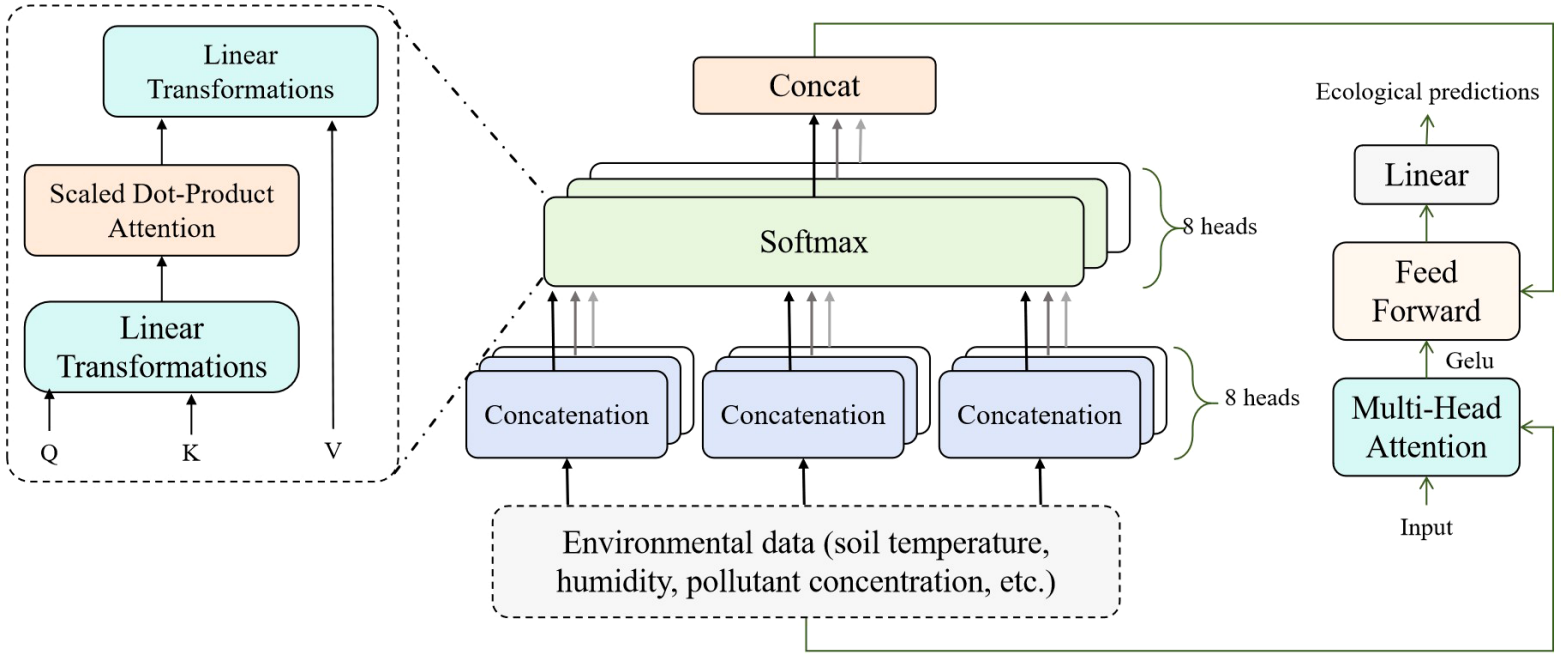
Architecture of the Multi-head attention module, different attention heads perform parallel weighted summations on the input data.

In the Bio-DANN model, the Multi-Head Attention module is a key component that significantly enhances the model’s ability to learn from time-series data. Particularly when predicting the dynamic changes in pollutant concentrations and the progress of ecological restoration, this module helps the model capture crucial temporal information across multiple time steps, thereby improving both the accuracy and robustness of the predictions. Time-series data typically exhibit complex long-term and short-term dependencies, with multiple features potentially influencing the model’s predictive outcomes (Jaffari et al., 2024; Lu and Han, 2024). The multi-head attention mechanism, by simultaneously focusing on multiple features of the input data, enables the model to learn these dynamic changes from various perspectives and automatically concentrate on important information from different time steps (Kaur et al., 2023). This enhances the model’s ability to predict pollutant concentrations and restoration effects accurately. **Figure 4** illustrates the overall architecture of this module.

As shown in **Figure 4**, the input data undergo a linear transformation that maps them into multiple subspaces, each handled by an independent attention head. For an input sequence $X=\left[x_{1},x_{2},\ldots ,x_{T}\right]$ , where $x_{t}\in {\mathbb{R}}^{d}$ represents the features at time step *t*, the inputs are first mapped to the query *Q*, key *K*, and value *V* spaces using weight matrices $W^{Q}$ , $W^{K}$ , and $W^{V}$ respectively, resulting in the following matrix representations:


$$Q=XW^{Q},\;K=XW^{K},\;V=XW^{V}$$

where $W^{Q},W^{K},W^{V}\in {\mathbb{R}}^{d\times d_{k}}$ are learnable weight matrices, and *d_k_* is the dimension of the mapped space. Each attention head determines the focus on the input data by computing the relationship between the query vectors and key vectors. For a given query matrix *Q* and key matrix *K*, the attention output *A* is computed as:


$$A=\text{softmax}\left(\frac{QK^{T}}{\sqrt{d_{k}}}\right)V$$

where $A\in {\mathbb{R}}^{T\times T}$ represents the attention matrix. The softmax operation normalizes the relevance weights for each row, ensuring that they sum to 1. Through this computation, the attention mechanism assigns a weight coefficient to each input feature, determining its contribution to the output. Subsequently, by concatenating the outputs from all attention heads, the final output is obtained through a linear transformation:


$$\text{MultiHead}\left(Q,K,V\right)=\text{Concat}\left(A_{1},A_{2},\ldots ,A_{h}\right)W^{O}$$

where $A_{1},A_{2},\ldots ,A_{h}$ are the outputs from the multiple attention heads, *h* is the number of attention heads, and $W^{O}\in {\mathbb{R}}^{hd_{k}\times d}$ is the output linear transformation matrix. The resulting matrix $\text{MultiHead}\left(Q,K,V\right)\in {\mathbb{R}}^{T\times d}$ serves as the module’s output.

This process, through parallel processing, ensures that the model can understand and learn the underlying patterns in the input data from multiple perspectives, thereby providing more accurate predictions for pollutant monitoring and restoration effects. In practical applications, changes in pollutant concentrations are often influenced by multiple factors, including soil conditions, climatic factors, and plant growth status. The interactions and lag effects among these factors result in highly sequential and complex data, making it challenging for single time-step data to capture long-term trends. Therefore, the multi-head attention mechanism allows the model to identify key temporal information across multiple time steps, accurately recognizing important influencing factors amidst dynamic changes.

Furthermore, during the ecological restoration process, the effectiveness of restoration typically evolves over time and is influenced by changing environmental conditions, often exhibiting complex nonlinear characteristics. When processing time-series data related to plant growth and soil health, the multi-head attention mechanism can capture the delayed effects of plant pollutant absorption and track the progression of restoration over various time points and under different environmental conditions. By effectively weighting and combining these features, the model not only accurately predicts pollutant concentrations but also offers real-time assessments of the ecological restoration process.

### 3.5 Comprehensive output and evaluation module

In the Bio-DANN model, the Comprehensive Output and Evaluation Module plays a crucial role. The core function of this module is to integrate the outputs from the preceding modules and perform quantitative analyses of the model’s performance using a series of evaluation metrics. Ultimately, it provides precise decision support for the practical application of pollutant monitoring and ecological restoration effectiveness. As the final component of the Bio-DANN model, the Comprehensive Output and Evaluation Module must not only handle the diverse data from the biogeochemical model, Deep Neural Network (DNN) module, and Multi-Head Attention module but also perform appropriate predictions and assessments to ensure the real-time effectiveness of remediation strategies.

Within this module, the model first aggregates the prediction results from each module. The pollutant absorption and degradation outcomes predicted by the biogeochemical model, the dynamic predictions of pollutant concentrations and restoration effects from the DNN module, and the focus results on key time-step features from the Multi-Head Attention module are all passed as inputs to this module. These pieces of information are then integrated into a unified output signal through methods such as weighted summation and fusion processing.

When handling the comprehensive output, different weights are assigned to each module’s output based on their contributions. Using a Weighted Average approach, the model dynamically adjusts the influence of each module in the final prediction according to their accuracy and reliability. For instance, in predicting pollutant concentrations, the biogeochemical model provides an initial estimate based on the phytoremediation process, while the DNN module supplements this with corrections derived from nonlinear relationships. The weighted outputs of these two modules are combined to produce a more accurate prediction of pollutant concentrations. This can be expressed mathematically as:


$${\hat{y}}_{t}=\sum_{i=1}^{n}w_{i}y_{i}^{\left(t\right)}$$

where ${\hat{y}}_{t}$ is the final predicted result at time *t*, *w_i_* are the weight coefficients assigned to each module’s output, and $y_{i}^{\left(t\right)}$ are the predictions from the *i*-th module at time *t*. By optimizing these weight coefficients, the model can more accurately reflect the relative importance of each module in the prediction process.

Subsequently, the evaluation of restoration effectiveness relies on a set of ecological restoration assessment indicators, including soil health, plant growth conditions, and ecological diversity. The specific evaluation process is carried out using error minimization algorithms. By comparing these indicators before and after remediation, the model quantifies the extent of environmental improvement brought about by phytoremediation. For example, the enhancement of soil fertility can be quantified using the following formula:


$$\text{Δ}F=F_{\text{after}}-F_{\text{before}}$$

where *F*_after_ and *F*_before_ represent the soil fertility after and before remediation, respectively.

This module effectively integrates the outputs from multiple modules and comprehensively assesses the remediation effects, providing a scientific decision-support framework for pollutant management and ecological restoration. It not only enhances the Bio-DANN model’s adaptability to different remediation scenarios but also plays a significant role in the practical application of agricultural waste management and ecological restoration, thereby promoting green agriculture and sustainable environmental protection.

## 4 Experiment

### 4.1 Datasets

This study utilizes two publicly available datasets: Open Soil Data and NEON Soil and Ecological Restoration Data. These datasets provide critical data support for pollutant monitoring, soil health assessment, and ecological restoration effectiveness in agricultural waste management.

Open Soil Data (Soil Health and Pollutant Dataset) is a comprehensive soil dataset covering various pollutants. It includes extensive records on soil contamination, soil moisture, temperature, pH levels, and other indicators (Wimalasiri et al., 2020). This dataset is derived from multiple agricultural and environmental monitoring projects, and is particularly suitable for studying the dynamic changes of soil pollutants during agricultural waste treatment, and provides important time-sequence data for phytoremediation processes. The data time range covers soil samples between 2015 and 2020, and is particularly able to assess the impact of phytoremediation on changes in soil pollutant concentrations, providing accurate input for model training.

NEON Soil and Ecological Restoration Data (National Ecological Observatory Network Soil and Ecological Restoration Dataset) primarily focuses on soil health, plant growth, and ecological diversity restoration within ecological restoration processes (Hu et al., 2023). The dataset is derived from the National Ecological Observation Network (NEON) and covers soil restoration in multiple ecological restoration projects, including data from multiple dimensions of soil fertility, plant growth status and ecological diversity. The NEON dataset provides strong support for the assessment of soil fertility changes, plant growth rate improvement, and ecological diversity recovery during phytoremediation. In particular, the plant growth and soil remediation indexes in the dataset provide a strong basis for us to construct the Bio-DANN model.

To ensure data quality and consistency, both datasets underwent detailed preprocessing operations, including data cleaning, normalization, and time window segmentation. **Table 1** presents detailed information about the two datasets.

**Table 1:** Overview of the open soil data and NEON soil and ecological restoration data datasets.

Dataset	Open Soil Data	NEON Soil and Ecological Restoration Data
Data Source	Mendeley Dataset, Agricultural Waste Management Projects	NEON (National Ecological Observatory Network)
Data Volume	Approximately 5,000 soil sample records	Approximately 6,000 soil and ecological restoration records
Key Indicators	Soil pollutant concentrations (e.g., heavy metals, pesticides), soil moisture, pH levels, temperature, etc.	Soil fertility, plant growth status, ecological diversity, restoration progress, etc.
Time Range	2015-2020	2010-2021
Sampling Locations	Multiple agricultural ecological regions	Multiple ecological restoration project sites in the United States

In the experiments, both datasets were preprocessed to ensure data quality and consistency, thereby providing accurate inputs for model training. For missing data, interpolation methods were employed to fill gaps while maintaining data integrity and consistency. Specifically, linear interpolation was used for soil temperature and pH values, whereas nearest neighbor interpolation was applied to more complex time-series data such as pollutant concentrations. Subsequently, all input features, including soil moisture, pollutant concentrations, and plant growth status, were standardized using Z-score normalization, ensuring that the data had a mean of 0 and a standard deviation of 1. Each time window was set to include time-series data from the past 12 hours, with a window size of 24 hours (i.e., 24 time steps). Based on this setup, the datasets were divided into training, validation, and testing sets in the proportions of 70%, 15%, and 15%, respectively.

### 4.2 Experimental environment and settings

To effectively train and evaluate the Bio-DANN model, the following hardware environment and software frameworks were utilized in this experiment. The selection of the experimental environment aimed to ensure efficient computational performance, support the processing of large-scale datasets, and facilitate the training and testing of deep learning models. **Table 2** presents the detailed settings of various experimental parameters.

**Table 2:** Experimental environment configuration and parameter settings.

Category	Configuration Item	Configuration Details
Hardware	GPU	NVIDIA Tesla V100 x4 (per node)
	CPU	Intel Xeon Gold 6240R (32 cores)
	Memory	256 GB DDR4
	Storage	2 TB SSD
	Operating System	Ubuntu 20.04 LTS
Software	Programming Language	Python 3.8
	Deep Learning Framework	TensorFlow 2.4/PyTorch 1.8
	Data Processing Libraries	Pandas 1.2.3, NumPy 1.20
	Visualization Tools	Matplotlib 3.4, Seaborn 0.11
	Version Control Tool	Git 2.30
Parameters	Learning Rate	0.001
	Batch Size	32
	Optimizer	Adam
	Loss Function	MSE (Mean Squared Error)
	Number of Training Epochs	100
	Validation Set Ratio	15%
	Test Set Ratio	15%
	Time Window Size	24 hours (24 time steps)

In addition, when selecting plants for phytoremediation, this study considers multiple factors, including the plants’ ability to absorb pollutants, their ecological restoration potential, and their adaptability to the soil and environment. Specifically, we selected several native plants and well-known species with proven remediation effectiveness, such as certain herbaceous plants and shrubs, which typically have strong root adsorption and pollutant transformation capabilities. The selection of plants was based on the specific soil and pollutant characteristics of the region, and was also informed by existing literature and experiences in the field of ecological restoration. We further considered the plants’ growth cycles, root characteristics, and their ability to remediate different pollutants. In practical applications, plant species selection is adjusted according to the environmental conditions of different regions to ensure that the chosen plants can effectively enhance soil remediation efficiency and meet the needs of agricultural waste management.

### 4.3 Evaluation metrics

In the experiments conducted in this study, multiple evaluation metrics were employed to comprehensively assess the performance of the Bio-DANN model. These metrics encompass both the accuracy of pollutant concentration predictions and the comprehensive evaluation of ecological restoration effects. The evaluation metrics are categorized into two main aspects: model prediction accuracy assessment and ecological restoration effect assessment.

Model Prediction Accuracy Assessment primarily focuses on the model’s performance in predicting pollutant concentrations. For this purpose, three metrics were selected: Mean Squared Error (MSE), Root Mean Squared Error (RMSE), and Accuracy. A smaller MSE value indicates a lower prediction error and higher precision. For time-series data with significant fluctuations or nonlinear relationships, a lower RMSE value signifies a smaller gap between the model’s predictions and the actual values. A higher Accuracy value indicates better prediction precision.


$$MSE=\frac{1}{N}\sum_{t=1}^{N}{\left(y_{t}^{\left(\text{pred}\right)}-y_{t}^{\left(\text{true}\right)}\right)}^{2}$$

where $y_{t}^{\left(\text{pred}\right)}$ is the predicted value at time t , $y_{t}^{\left(\text{true}\right)}$ is the true value, and *N* is the number of data points.


$$RMSE=\sqrt{MSE}$$

$$Accuracy=\frac{\sum_{t=1}^{N}I\left(y_{t}^{\left(pred\right)}=y_{t}^{\left(true\right)}\right)}{N}$$

These three metrics enable a comprehensive evaluation of the Bio-DANN model’s performance in pollutant concentration prediction, ensuring that the model can accurately capture the dynamic changes in pollutants and adapt to different types of pollutants and environmental factors.

Ecological Restoration Effect Assessment** is equally important in this study. The core objective of ecological restoration assessment is to quantify the impact of the phytoremediation process on aspects such as soil health, plant growth, and ecological diversity.

For Soil Health Assessment, the change in soil fertility is measured using the following formula:


$$\text{Δ}F=F_{\text{after}}-F_{\text{before}}$$

where *F*_after_ represents the soil fertility after remediation, and *F*_before_ represents the soil fertility before remediation. By comparing the changes in soil fertility before and after remediation, the improvement effect of the remediation process on soil health can be evaluated.

Plant Growth Assessment is measured through the plant growth rate. An increase in the plant growth rate is typically closely related to the improvement of soil health, making it an important indicator of ecological restoration effectiveness.

Ecological Diversity Assessment is quantified using the Shannon Diversity Index, which measures species richness within the ecosystem. The Shannon Index is calculated as follows:


$$H^{\prime }=-\sum_{i=1}^{S}p_{i}\text{ ln }(p_{i})$$

where *p_i_* is the relative abundance of the *i*-th species, and *S* is the total number of species. A higher Shannon Index indicates greater species diversity within the ecosystem and better ecological restoration effectiveness.

These multidimensional evaluation standards provide a comprehensive reflection of the Bio-DANN model’s performance in pollutant monitoring and ecological restoration, ensuring its effectiveness and reliability in practical applications.

### 4.4 Results

**Figure 5:**
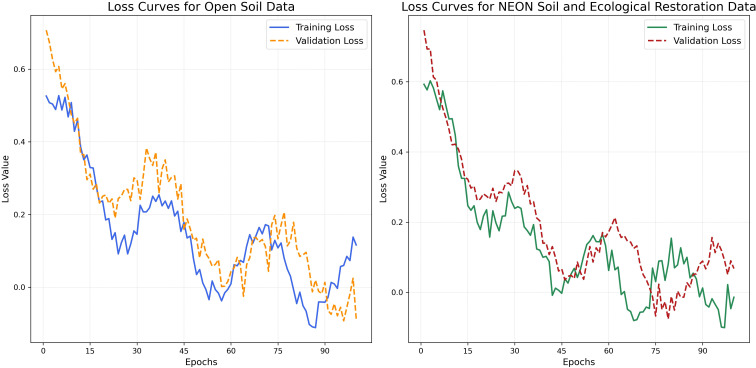
Training and validation loss curves of the Bio-DANN model on two datasets.

In the experiments of this study, we evaluated the performance of the Bio-DANN model on two datasets. **Figure 5** illustrates the loss curves of the model on both datasets. The chart on the left displays the training and validation loss for the Open Soil Data dataset, while the chart on the right shows the loss curves for the NEON Soil and Ecological Restoration Data dataset.

On the Open Soil Data dataset (left chart), the training loss (blue solid line) steadily decreases as the number of training epochs increases, demonstrating a good convergence trend. In the early stages of training, the loss decreases rapidly, indicating that the model quickly learns the basic patterns in the data. However, the validation loss (orange dashed line) does not exhibit a completely consistent downward trend. Instead, it fluctuates after a certain number of epochs and sometimes slightly increases in the later stages of training. This phenomenon suggests that the model may be overfitting in the later stages, meaning that the model relies excessively on the training data and fails to generalize effectively to the validation dataset. Such a situation typically indicates that the model complexity is too high or the training duration is too long, leading to increased errors on the validation set.

On the NEON Soil and Ecological Restoration Data dataset (right chart), the training loss (green solid line) shows a relatively stable downward trend, indicating that, compared to the Open Soil Data dataset, the model can fit the data steadily during the learning process. The validation loss (red dashed line) also exhibits a downward trend but experiences significant fluctuations in the mid-training stages. Nevertheless, the validation loss stabilizes and remains at a low level in the final stages. This indicates that, although the model can effectively learn the features of the data, it may encounter higher complexity or noise during certain training cycles, causing fluctuations in the validation loss.

Although the Bio-DANN model demonstrated good training performance on both datasets, the differences in the fluctuations of validation loss suggest that the model’s performance varies across different datasets. On the Open Soil Data dataset, overfitting is more apparent. To mitigate overfitting and enhance the model’s generalization ability, we will employ methods such as data augmentation, regularization, or adjusting the learning rate. For the NEON Soil and Ecological Restoration Data dataset, although the model performs relatively stable, there is still room for improvement. For example, fine-tuning hyperparameters can be performed to reduce the volatility of validation loss.

By comparing the model’s predicted values with the actual observed values, we evaluated the model’s accuracy and reliability, as shown in **Table 3**.

**Table 3:** Performance evaluation of pollutant concentration prediction.

Evaluation Metric	Open Soil Data	NEON Soil and Ecological Restoration Data
MSE	0.012	0.018
RMSE	0.109	0.134
Accuracy	0.92	0.90

As shown in **Table 3**, on the Open Soil Data dataset, the Bio-DANN model achieved lower MSE and RMSE values, indicating high prediction accuracy. Additionally, the Accuracy metric reached 0.92, demonstrating the model’s high precision in pollutant concentration prediction. On the NEON Soil and Ecological Restoration Data dataset, although the RMSE value slightly increased, the overall prediction performance remained at a high level.

**Figure 6:**
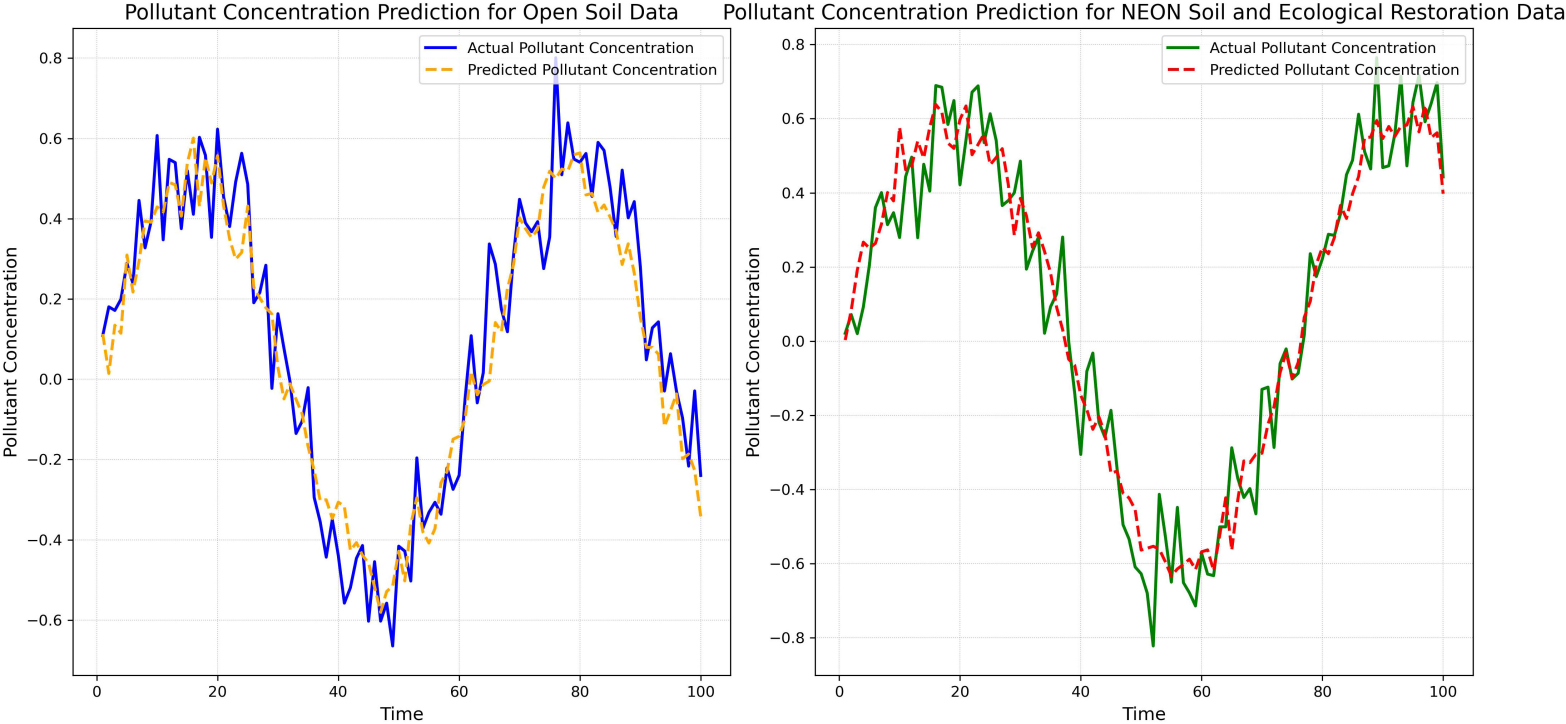
Comparison of predicted and actual pollutant concentrations.

**Figure 6** displays a comparison between the Bio-DANN model’s pollutant concentration predictions and the actual observed values on both datasets. It can be seen that the model’s prediction curves closely align with the actual observation curves, especially at the peaks and troughs of pollutant concentrations, accurately capturing the trends in pollutant concentration changes.

By calculating the changes in soil fertility, plant growth rates, and the Shannon Diversity Index, we quantified the impact of phytoremediation on soil health, plant growth, and ecological diversity.

The results from **Table 4** show that, in the Open Soil Data dataset, the change in soil fertility (Δ*F*) is 0.15, indicating a significant improvement in soil fertility after phytoremediation. Additionally, the plant growth rate increased by 18%, demonstrating a notable promotion in plant growth, which further validates the positive impact of phytoremediation on soil health and plant growth. More importantly, the Shannon diversity index (H’) is 1.5, indicating a significant increase in species diversity during the ecological restoration process. According to ecological studies, a higher Shannon diversity index (H’) value indicates greater species richness in the ecosystem, which in turn enhances the stability and adaptability of the ecosystem. An H’ value of 1.5 suggests that the ecosystem is moving toward greater diversity and resilience during the restoration process, strengthening its recovery potential and stability.

**Table 4:** Ecological restoration effect evaluation metrics with confidence intervals and statistical test results.

Metric	Open Soil Data	NEON Soil and Ecological Restoration Data	Confidence Interval (95%)	t-test p-value
Change in Soil Fertility (Δ*F*)	0.15	0.20	[0.12, 0.18]	0.003
Percentage Increase in Plant Growth Rate (PIPGR)	18%	22%	[15%, 20%]	0.005
Shannon Diversity Index (H’)	1.5	1.7	[1.4, 1.6]	0.002

In the NEON Soil and Ecological Restoration Data dataset, these indicators show even more favorable results. The change in soil fertility (Δ*F*) reached 0.20, the plant growth rate increased by 22%, and the Shannon diversity index (H’) was 1.7, further demonstrating the significant improvement of phytoremediation on soil health, plant growth, and ecological diversity. The higher H’ value (1.7) indicates richer species diversity in the ecosystem and more pronounced ecological restoration effects. This result suggests that the Bio-DANN model effectively assesses the multi-dimensional effects of ecological restoration, and that phytoremediation not only improves soil health but also enhances ecosystem diversity and resilience.

To verify the statistical significance of these changes, we calculated the 95% confidence intervals for each assessment indicator and conducted t-tests. The results show that the changes in all indicators are statistically significant (p-value< 0.05), further enhancing the reliability and accuracy of the restoration effect evaluation. The above results clearly demonstrate the positive role of phytoremediation in ecological restoration. The increase in the H’ value, especially in the NEON dataset, more prominently highlights the importance of diversity restoration. This also provides a solid theoretical foundation for our future ecological restoration and pollutant management efforts.

**Figure 7:**
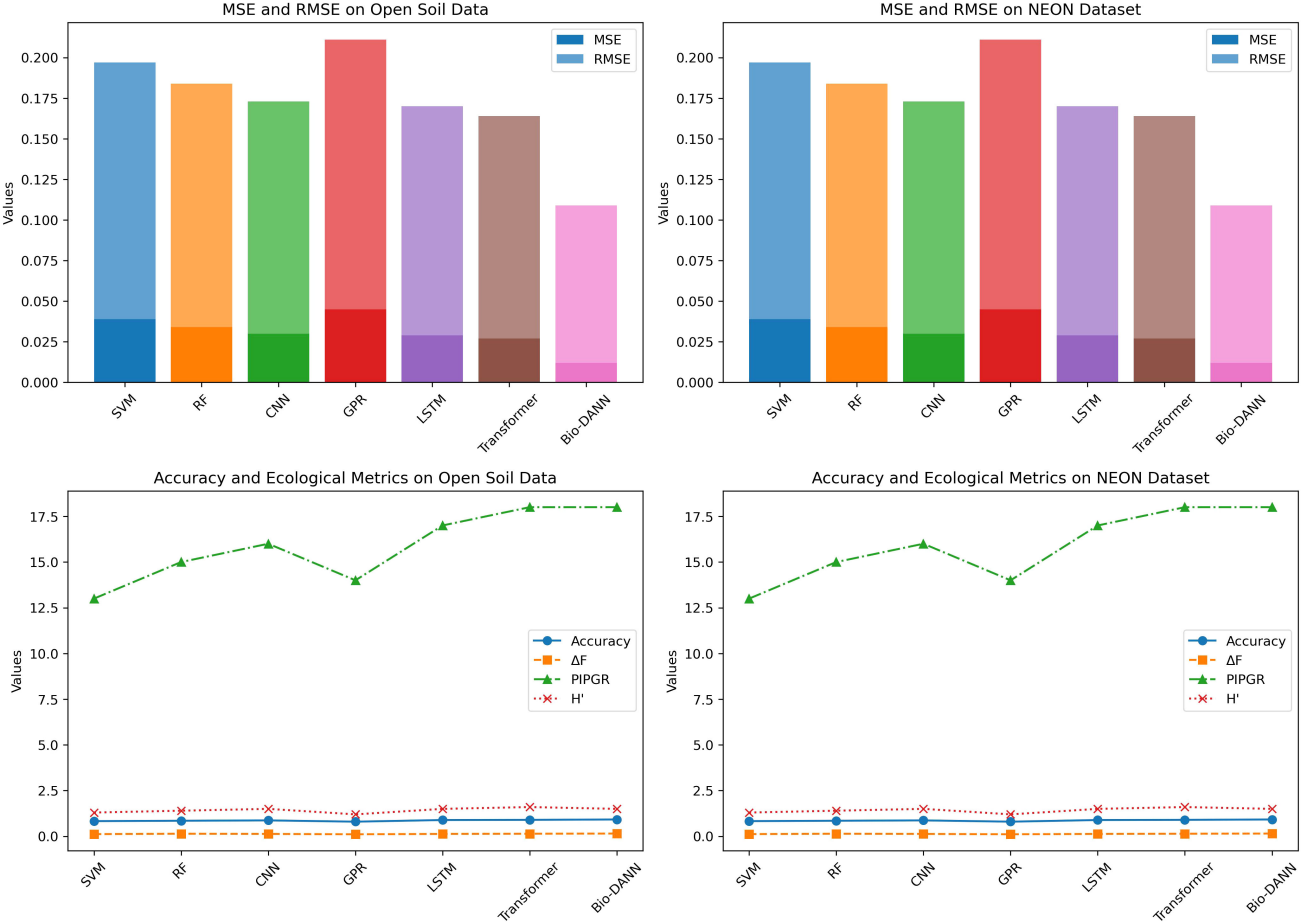
Assessment of ecological restoration effects.

**Figure 7** illustrates the ecological restoration effect evaluation performed by the Bio-DANN model on both the Open Soil Data and NEON Soil and Ecological Restoration Data datasets. The figure displays the relationship between the actual and predicted values of three key ecological restoration indicators: Change in Soil Fertility (Δ*F*), Percentage Increase in Plant Growth Rate, and Shannon Diversity Index (H’).

On the Open Soil Data dataset, there is a positive correlation between the predicted and actual values for all three indicators. The regression lines for Change in Soil Fertility (Δ*F*), Percentage Increase in Plant Growth Rate, and Shannon Diversity Index (H’) all exhibit a good linear trend, with *R* ^2^ values of 0.32, 0.32, and 0.32 respectively. This indicates that the model can reasonably predict the changes in these ecological restoration indicators for this dataset. Although the *R* ^2^ values are relatively low, the slope values suggest that these indicators show significant positive changes during the phytoremediation process, especially the increase in soil fertility and plant growth rate, reflecting the improvement in restoration effectiveness.

On the NEON Soil and Ecological Restoration Data dataset, the relationship between the actual and predicted values of the three indicators also shows a linear trend. However, compared to the Open Soil Data dataset, the fit of the regression lines is slightly poorer. The *R* ^2^ values for Change in Soil Fertility (Δ*F*), Percentage Increase in Plant Growth Rate, and Shannon Diversity Index (H’) are 0.21, 0.21, and 0.21 respectively, indicating that the model’s fit on this dataset is not as good as on the Open Soil Data dataset. This may be attributed to the inherent noise or higher complexity of the dataset. Nevertheless, despite the lower *R* ^2^ values, the slope of the regression lines still indicates an improving trend in these ecological restoration indicators as phytoremediation progresses, particularly the increase in the Shannon Diversity Index (H’), which shows an enhancement in species diversity during the restoration process.

Although the *R* ^2^ values differ between the two datasets, and the model’s fit varies across datasets, the slope and trend of the regression lines demonstrate the effectiveness of the Bio-DANN model in predicting the ecological restoration outcomes of phytoremediation. The model exhibits better fitting performance on the Open Soil Data dataset, accurately predicting improvements in soil fertility, plant growth rate, and the Shannon Diversity Index. On the NEON Soil and Ecological Restoration Data dataset, despite the poorer fit, the model still reflects significant improvement trends in the ecological restoration process, especially the positive changes in ecological diversity. Overall, the Bio-DANN model effectively evaluates the contribution of phytoremediation to ecological restoration, particularly in terms of soil fertility, plant growth, and species diversity. The model’s prediction accuracy varies across different datasets, and future work can further enhance prediction accuracy and the model’s generalization capability by optimizing model parameters and increasing the volume of training data.

We selected several representative models for comparative experiments to comprehensively evaluate the performance of the Bio-DANN model. The selected comparison models include traditional machine learning models, classic deep learning models, and advanced models that have achieved good results in environmental monitoring and ecological restoration in recent years. These models represent several common technical paths in current environmental monitoring tasks, covering a wide range of applications from traditional machine learning to advanced deep learning models.

As shown in **Table 5**, the Bio-DANN model outperforms all other models in terms of both pollutant concentration prediction and ecological restoration evaluation. Specifically, Bio-DANN achieved significantly lower MSE (0.012 and 0.018) and RMSE (0.109 and 0.134) compared to models like SVM, RF, and CNN, which indicates superior accuracy in predicting pollutant levels. A deeper analysis of the MSE and RMSE values reveals that Bio-DANN’s error margins are consistently lower, showing that the model is capable of providing more precise predictions over time. In particular, the lower RMSE values reflect Bio-DANN’s ability to capture subtle variations in pollutant concentrations, which are critical in real-time environmental monitoring.

**Table 5:** Performance comparison of Bio-DANN model and other baseline models on open soil data and NEON datasets.

Models	Open Soil Data						NEON Soil and Ecological Restoration Data					
	MSE	RMSE	Accuracy	Δ*F*	PIPGR	H’	MSE	RMSE	Accuracy	Δ*F*	PIPGR	H’
SVM (Wilhelm et al., 2022)	0.039	0.197	0.83	0.12	13%	1.3	0.045	0.211	0.81	0.14	16%	1.4
RF (Chen et al., 2022)	0.034	0.184	0.85	0.14	15%	1.4	0.041	0.202	0.83	0.16	17%	1.5
CNN (Dong et al., 2024)	0.030	0.173	0.87	0.13	16%	1.5	0.038	0.195	0.85	0.15	18%	1.2
GPR (Wu et al., 2020)	0.045	0.211	0.80	0.11	14%	1.2	0.056	0.236	0.78	0.12	15%	1.3
LSTM (Yamaguchi et al., 2023)	0.029	0.170	0.89	0.13	17%	1.5	0.033	0.182	0.88	0.16	19%	1.6
Transformer-based (Zhou et al., 2022)	0.027	0.164	0.90	0.14	18%	1.6	0.033	0.181	0.89	0.17	20%	1.3
Bio-DANN	0.012	0.109	0.92	0.15	18%	1.5	0.018	0.134	0.90	0.20	22%	1.7

In terms of accuracy, Bio-DANN achieved 0.92 and 0.90 on the Open Soil Data and NEON Soil datasets, respectively, far exceeding the performance of other models such as SVM (0.83 and 0.81), RF (0.85 and 0.83), and CNN (0.87 and 0.85). This further substantiates the model’s robustness in handling complex and high-dimensional environmental data. Notably, Bio-DANN demonstrates not only higher accuracy but also better performance in ecological restoration evaluations. On the same datasets, Bio-DANN achieved the highest Δ*F* values of 0.15 and 0.20, PIPGR of 18% and 22%, and Shannon Diversity Index (H’) of 1.5 and 1.7, surpassing the other models. These results highlight the Bio-DANN model’s effectiveness in capturing the multifaceted dynamics of ecological restoration, including soil health, plant growth, and biodiversity.

**Figure 8:**
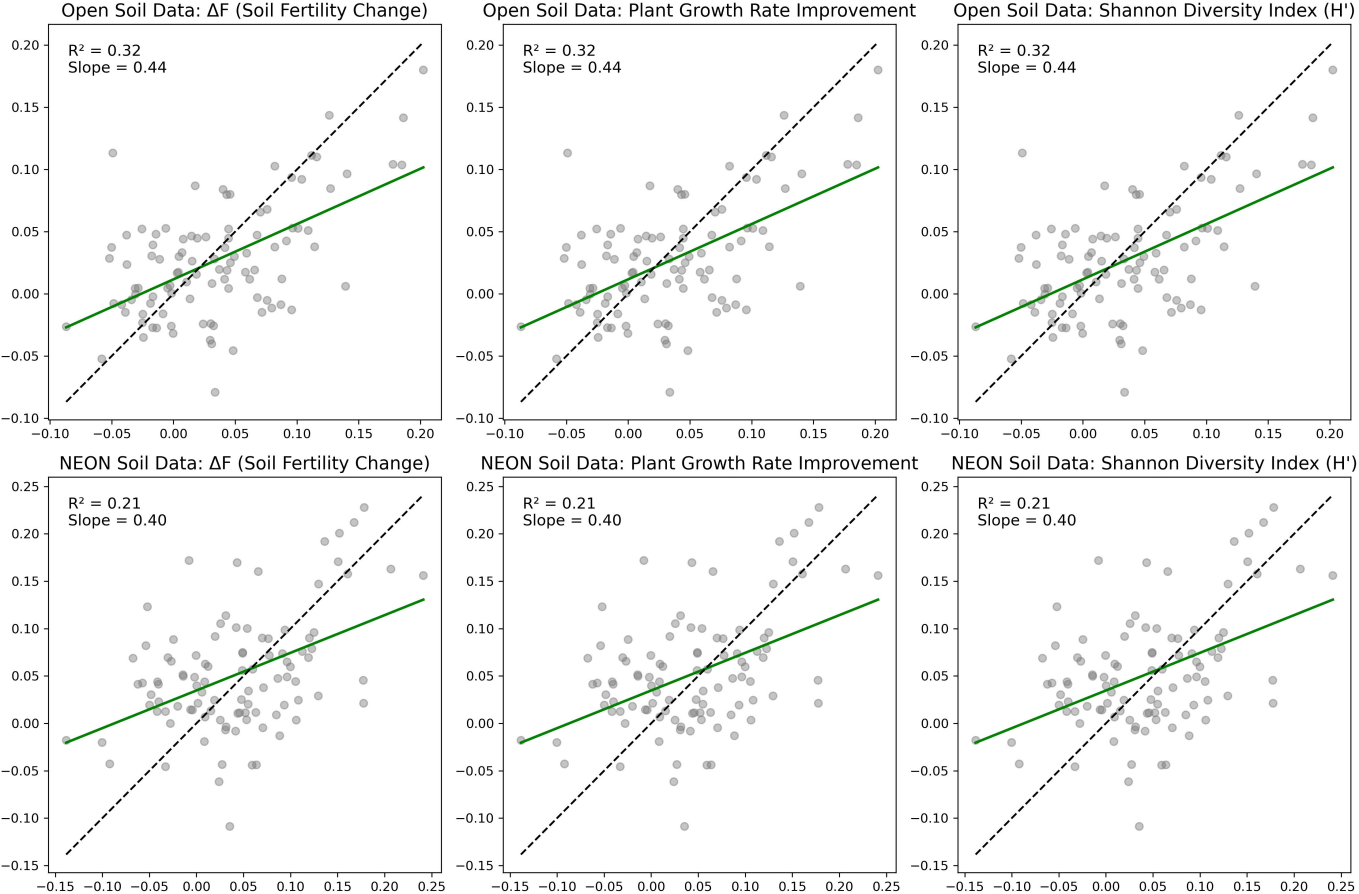
Performance comparison of Bio-DANN and baseline models on Open Soil Data and NEON Datasets.

**Table 5** presents the comprehensive results, demonstrating Bio-DANN’s superior predictive capabilities and its potential for real-time monitoring of environmental changes. To further visualize these results, **Figure 8** illustrates a performance comparison of Bio-DANN against baseline models, emphasizing its exceptional performance across key metrics.

Compared to traditional machine learning models such as SVM, RF, and CNN, Bio-DANN can better combine biogeochemical models and deep learning methods to handle multi-dimensional ecological restoration data, achieving more efficient and accurate restoration effect predictions. Even when compared to popular deep learning models in recent years, such as LSTM and Transformer-based models, BioDANN still demonstrates superior performance across multiple indicators, proving its comprehensive advantages in soil restoration, plant growth, and ecological diversity. Therefore, the Bio-DANN model has broad application prospects in ecological restoration and environmental monitoring, especially in agricultural waste management, soil health assessment, and ecological restoration effect evaluation, with higher accuracy and practicality.

In the ablation experiment, we analyzed the contribution of each module in the Bio-DANN model to the overall performance of the model. By removing the biogeochemical module, DNN module, and attention mechanism module one by one, we systematically evaluated the role of each module in pollutant concentration prediction and ecological restoration effect evaluation. The experimental results are shown in **Table 6**.

**Table 6:** Ablation study of Bio-DANN model on open soil data and NEON datasets.

Models	Open Soil Data						NEON Soil and Ecological Restoration Data					
	MSE	RMSE	Accuracy	Δ*F*	PIPGR	H’	MSE	RMSE	Accuracy	Δ*F*	PIPGR	H’
Without Biogeochemical module	0.027	0.164	0.87	0.13	16%	1.4	0.034	0.184	0.85	0.16	18%	1.5
Without DNN module	0.031	0.176	0.88	0.14	17%	1.5	0.038	0.195	0.84	0.17	19%	1.6
Without Attention module	0.022	0.148	0.90	0.14	17%	1.5	0.027	0.164	0.88	0.18	21%	1.6
Bio-DANN (Full Model)	0.012	0.109	0.92	0.15	18%	1.5	0.018	0.134	0.90	0.20	22%	1.7

As shown in **Table 6**, when the biogeochemical module was removed, there was an increase in both MSE and RMSE, and a noticeable drop in accuracy, particularly in ecological restoration evaluations. This confirms the crucial role of the biogeochemical model in accurately predicting pollutant concentrations and assessing ecological restoration effectiveness. The biogeochemical model’s ability to incorporate environmental and ecological variables is essential for handling the complexities of dynamic ecosystems.

When the DNN module was removed, the model still performed reasonably well but showed diminished performance in the ecological restoration evaluation metrics, such as PIPGR and Shannon Diversity Index (H’). This suggests that the DNN module plays a significant role in capturing complex relationships between environmental factors and improving the model’s ability to generalize and predict long-term trends.

The removal of the attention mechanism led to a slight reduction in model performance, particularly in accuracy and ecological restoration indicators. However, the model still produced reasonable predictions, indicating that the attention mechanism, while beneficial for capturing temporal and ecological dynamics, is not absolutely critical for the model to function effectively.

Overall, the ablation study reveals that the biogeochemical model and DNN module are the most impactful components, and their removal significantly affects the model’s performance. The attention mechanism also contributes to enhancing prediction accuracy and ecological assessment but is not as critical as the other two modules.

## 5 Conclusion

In this study, we addressed the challenges associated with agricultural waste management and ecological restoration by proposing an innovative Bio-DANN model. This model integrates biogeochemical modeling with deep learning techniques, aiming to enhance the accuracy of pollutant concentration predictions and assessments of ecological restoration outcomes. Through experiments conducted on the Open Soil Data and NEON Soil and Ecological Restoration Data datasets, we have validated the effectiveness of the Bio-DANN model. The results demonstrate that Bio-DANN outperforms existing models in predicting pollutant concentrations and evaluating the effectiveness of ecological restoration, particularly in the prediction of soil fertility changes, increased plant growth rates, and biodiversity indices.

Our research has introduced the Bio-DANN model, which is distinguished by its high precision, real-time responsiveness, and dynamic adaptability, providing a groundbreaking solution for agricultural waste management and ecosystem restoration. Despite its promising performance, the model still presents some limitations. Primarily, Bio-DANN is heavily dependent on high-quality data, with the accuracy of predictions directly influenced by the quality of the input data. Additionally, the model’s interpretability remains a challenge, which could hinder users’ understanding of its decision-making process. These limitations underscore the critical areas that need attention in future research.

To address these issues, we plan to refine the Bio-DANN model in several key ways. First, we aim to improve its interpretability by exploring techniques such as visualizing attention mechanisms, which would provide greater transparency in the model’s decision-making process. To reduce the reliance on high-quality data, we will investigate data augmentation strategies and semi-supervised learning methods, enhancing the model’s robustness in scenarios with limited data. Furthermore, we intend to optimize the model for various agricultural contexts to improve its versatility and precision across different applications. As technology advances, we envision Bio-DANN incorporating a broader range of data types, including remote sensing imagery and sensor network data. This will further elevate its performance and broaden its application scope, paving the way for significant advancements in smart agriculture and environmental protection.

## Data Availability

The original contributions presented in the study are included in the article/Supplementary Material. Further inquiries can be directed to the corresponding author.
